# Implementing AI innovation in radiology departments in the English NHS: a qualitative study on the experiences of professionals, patient groups and innovators

**DOI:** 10.3389/fdgth.2026.1736911

**Published:** 2026-03-17

**Authors:** Charitini Stavropoulou, Harry Scarbrough, Janette Rawlinson, Menghan Cui, David Baldwin, Nick Woznitza

**Affiliations:** 1 School of Health and Medical Sciences, City St George’s University of London, London, United Kingdom; 2 Centre for Health and Care Innovation Research, City St George’s University of London, London, United Kingdom; 3 Bayes Business School, City St George’s University of London, London, United Kingdom; 4 UK Lung Cancer and Mesothelioma Clinical Expert Group, Sandwell, United Kingdom; 5 UCL School of Management, University College London, London, United Kingdom; 6 Respiratory Medicine Unit, David Evans Centre, University of Nottingham, Nottingham, United Kingdom; 7 University College London Hospitals NHS Foundation Trust, London, United Kingdom; 8 School of Nursing, Midwifery, Allied & Public Health, Canterbury Christ Church University, Canterbury, United Kingdom; 9 Lungs for Living, University College London, London, United Kingdom

**Keywords:** artificial intelligence, implementation, lung cancer, NHS, radiology

## Abstract

**Introduction:**

Digital solutions and Artificial Intelligence (AI) innovations are often presented as the answer to many challenges faced by healthcare systems around the world. The UK government has made significant investments in this area, yet there have been concerns about the challenges faced when these technologies are implemented in practice. The aim of this study was to explore the perceptions and experiences of professionals, patient groups as well as innovators of introducing a new AI innovation used to detect potential abnormalities for lung cancer in radiology departments in the English NHS and to investigate the implementation challenges from their perspectives.

**Methods:**

Between September 2022 and January 2024, we visited five sites and conducted 34 interviews with radiologists, radiographers and other professionals involved in the implementation of the tool. We also interviewed seven staff from the company who developed and implemented the tool. In addition, three 2-hour focus group workshops, two online and one in person, were conducted in January 2024 with a total of 14 patient and public involvement and engagement (PPIE) participants from diverse regions, backgrounds and lived experience across England. Following initial coding done inductively, the Consolidated Framework for Implementation Research (CFIR) was applied as an organising framework to structure and interpret the emerging themes.

**Results:**

Both professional and PPIE groups recognised the potential of AI in the diagnostic pathway, while generally seeing it as a supportive second pair of eyes rather than an autonomous decision-maker, particularly when delivering sensitive news and information. Professionals’ acceptance depended on how the tool was integrated into existing workflows and its perceived value, with triaging functionality seen as effective, but varying in usefulness depending on local workload pressures. Innovators as well healthcare professionals highlighted a number of implementation challenges, particularly around fragmented legal and regulatory frameworks and unclear governance within the NHS.

**Discussion:**

Our findings underscore that successful AI implementation in clinical practice depends not on the technology alone but on its effective integration into existing healthcare contexts and alignment with the beliefs and needs of healthcare professionals, patients and the public.

## Introduction

Digital solutions and Artificial Intelligence (AI) innovations are key underpinning components of the National Health Service's (NHS's) operating model in which Government is making substantial investment (1, 2). These innovations, that often use algorithms to analyse medical images, automate tasks and personalise treatment, are seen as a solution to many clinical challenges (3), aiming to improve patient outcomes, reduce administrative burden and increase efficiencies. They have gained a lot of attention in areas such as radiology where the evidence on their impact on patient care is growing (4, 5) and where there is a need for early detection of cancer to improve cancer treatment outcomes (6).

Despite significant investments in new AI solutions and the Government's ambition to make the UK a leader in the AI field, the implementation and scaling up of these technologies remain limited in practice. A recent report published by the UK Parliament's Communications and Digital Committee warns that the UK risks becoming an “incubator economy” where innovative tech start-ups are developed locally but fail to scale up, calling for a more coherent industrial strategy (7).

Previous studies have cautioned against uncritical acceptance of the “hype” surrounding AI innovations (8), emphasising that significant translational challenges persist (9) and expressing doubts about the capacity of healthcare providers and professionals to successfully translate the potential of AI into reality (10). These challenges are not simply about datasets and infrastructure. They often relate to the human and behavioural aspects of translation, and the need for complementary changes in work practice (11). There is plenty of evidence to suggest that the views of healthcare professionals are key in determining the adoption of new technologies in practice and specifically when considering AI in healthcare settings (12). Equally, patients and their carers have their own views on how AI can be used in healthcare, which also determine their acceptance in practice (13). A recent study looking at the procurement and early implementation of AI tools in chest diagnostics in the UK revealed that despite its potential, integrating AI into clinical practice faces multiple challenges (14). These related to data quality, alignment with existing workflows, and the need for adequate staff training. The study also highlighted the difficulties of scaling AI solutions across diverse healthcare environments, emphasising the need for clear regulatory frameworks, standardised protocols and infrastructure enhancements. Similarly, Farič and colleagues explored the experiences of adopters of an AI-based imaging software used in radiology settings in the UK and showed that its performance was variable and call for more research on the socio-organizational factors that affecting (15).

With most of the focus on the technical capabilities of AI and its clinical outcomes, very little is known about the challenges faced when these technologies are deployed in practice. Some of the caution around introducing AI into healthcare reflects the need for robust evidence to support such change in practice. In the UK, the National Institute for Health and Care Excellence (NICE) has been conducting a number of Early Valuation Assessments on AI in healthcare, highlighting the need for more evidence to support its decisions prior to routine adoption within the NHS (16–18).

The aim of this study was to explore the perceptions and experiences of professionals, patients and innovators of introducing a new AI innovation in radiology departments in the English NHS and to investigate the implementation challenges from their perspectives.

## Materials and methods

Between September 2022 and January 2024 we conducted a qualitative study that included site visits, semi-structured individual interviews with healthcare professionals, staff and innovators, and workshops with patient and public involvement and engagement (PPIE) groups. Below we explain the empirical setting before providing more details regarding the methods we used to collect data from the different groups and the process we followed to analysed them.

### Empirical setting

The study was part of a prospective, multi-site, randomised trial (ISRCTN78987039), the primary aim of which was to investigate the impact of the immediate triage of chest x-rays using AI on the time to diagnosis of lung cancer (19). The AI tool, a [Conformité Européenne (CE)] approved medical device, detects and localises the presence of abnormalities on a chest x-ray. The output is generated in an automated manner for each chest x-ray in the form of a secondary capture identifying the region of interest and labelling of findings as well as prioritising abnormal cases for urgent interpretation. The tool had been tested in the past for its accuracy and was shown to have high sensitivity and specificity (20–22).

During the trial period, five days per fortnight (Monday to Friday) were block randomised to AI triage or routine care. Chest x-rays in the intervention group were processed by the AI tool and triaged for immediate report where findings suspicious of lung cancer were present. This AI decision tool was made available to the reporting radiographer or consultant radiologist when the immediate review was performed. Where appropriate, the patient was offered a same day chest computed tomography (CT) scan within a single diagnostic episode. The control group also had the AI output available at time of reporting but the chest x-rays were not prioritised in line with usual care.

The five radiology departments that deployed the tool included both general and university hospitals, and varied in terms of volume activity and geographical area, covering diverse patient populations. Only chest x-rays from primary care were included in the clinical trial. Inpatient and emergency chest x-rays were excluded.

### Data collection

#### Site visits and staff experiences

To capture staff perceptions and experiences we conducted a series of site visits and in-depth interviews in two stages. The pre-implementation stage included visits to all five sites before the implementation of the AI tool, between September 2022 and September 2023. During that stage we kept notes of all visits and conducted 11 in-depth interviews with hospital staff. The post-implementation stage included visits to the five sites that implemented the tool in their radiology departments. According to our notes, this was the first time an AI tool was deployed in each one of the radiology departments. We kept notes from each visit and conducted 23 interviews with hospital members who were involved in the implementation process. With the exception of two participants who had been involved with research on AI in radiology, all other participants had no previous experience with AI innovations. Table 1 presents the participants from each site, indicating the ones that were interviewed twice, prior to implementation and after deployment of the tool.

**Table 1:** Healthcare professionals interviews and key characteristics.

Participants	Sites	Key characteristics
P1 (interviewed twice)	Site_1	Consultant radiologist
P2 (interviewed twice)		Radiographer
P3		Radiographer
P4		Imaging IT lead
P5	Site_2	Consultant radiographer
P6		Consultant radiographer
P7		Radiographer
P8		Radiographer
P9		Systems manager
P10		Senior Clinical Systems Designer
P11		Radiographer
P12 (interviewed twice)	Site_3	Consultant radiologist
P13 (interviewed twice)		Research radiographer
P14		Radiographer
P15		Consultant radiographer
P16		Project manager R&D
P17 (interviewed twice)	Site_4	Consultant radiologist
P18		Radiographer clinical lead
P19		Radiographer
P20		Consultant radiologist
P21		Consultant radiologist
P22		Consultant radiologist
P23 (interviewed twice)	Site_5	Consultant radiologist
P24		Senior Clinical Research Practitioner
P25		Radiology Information Manager

The interviews were semi-structured, guided by questions and prompts in an interview guide based on previous literature and work that two researchers (HS and CS) had conducted in the past on the use of AI in radiology in the UK (12). The interview schedule can be found in the Supplementary Material. The interviews ranged from 25 to 110 min and were conducted in person during the site visits, or remotely if participants were unavailable on the day. The interviews were recorded then transcribed by an external professional company. We used purposive sampling to specifically target and identify users of the innovation at the implementation sites. More specifically, we first contacted the Principal Investigator (PI) of the NHS sites that were involved in the clinical trial and asked them to share the invitation to participate in our study with all staff involved in the deployment of the AI tool. Additionally, snowball sampling expanded the participant pool of end-users and other stakeholders from the sites by asking initial respondents to refer to other potential end-users. No incentives were offered to participants.

#### Innovators' perceptions and experiences

During the same period, we interviewed seven company staff, who were involved in the development and the implementation of the tool in practice. As shown in Table 2, they had different roles in the company allowing us to capture the perspectives and experiences from the innovators' perspectives.

**Table 2:** Company staff interviews and job roles.

Company staff	Job roles
I1	Chief of Staff/Operations
I2	Client Success
I3	Business Development UK
I4	UK Operations/Global Client Success
I5	Associate Director of Engineering
I6	Impact measurement
I7	Clinical research scientist
I8	Chief AI officer

Interviews were all conducted remotely via Microsoft Teams (Microsoft), recorded and then transcribed by an external professional company.

#### PPIE focus groups

The overall approach of seeking patient and carer experiences and views was discussed with the project's PPIE co-investigator (JR) and presented in two PPIE review panels at the Royal Marsden NHS Trust (London). Focus groups to explore PPIE views are used often in health research (23) and members of the research team had previous experience in using this method in exploring PPIE perceptions around the use of AI in clinical settings (24). A focus group schedule (see Supplementary Material) was co-developed between JR, CS and MC and shared with the PPIE review panel members for comments and feedback. It can be found in the Supplementary Material.

A flyer was designed and shared with PPIE fora, inviting patients who had a lung cancer diagnosis, as well as their friends and family members, to participate in the study and share their experiences of their diagnostic pathway and their perceptions around the use of AI in cancer diagnosis. Participants were reimbursed for their involvement in the study, following the guidance from the National Institute for Health and Care Research (NIHR's) (25).

In total, 14 participants were recruited. Table 3 presents their characteristics, showing a good balance between female and male participants, both patients and carers, from diverse regions across England and different diagnostic pathways. To allow more space for all voices to be heard, we organised small focus groups and offered participants the opportunity to choose between an in-person or online meeting. Three 2-hour focus group workshops were conducted in January 2024, comprising 2 online sessions hosted via Zoom, and 1 session conducted in person. The focus groups were facilitated by JR, CS and MC.

**Table 3:** PPIE workshop participant information list.

No.	Role	Location	Gender	Diagnosis	Year of diagnosis	Pathway
Q1	Patient	Oxford	Female	EGFR lung cancer	2022	Non-typical
Q2	Patient	Berkshire	Female	Lung cancer	2016	Non-typical
Q3	Carer	Leamington Spa	Female	Lung cancer	2019	Standard
Q4	Patient	Berkshire	Male	Ongoing checking	2017	Non-typical
Q5	Patient	Sanderstead	Female	Lung cancer	2019	Non-typical
Q6	Patient	Leicester	Female	Lung cancer	2019	Standard
Q7	Carer	London	Female	Non-small cell lung cancer	2020	Non-typical
Q8	Patient	Dorset	Female	Lung cancer	2005	Non-typical
Q9	Patient	Somerset	Female	Lung cancer	2021	Standard
Q10	Carer	Sutton	Male	Lung cancer	2022	Standard
Q11	Patient	Hull	Male	Lung sequestration	2017	Standard
Q12	Patient	Swindon	Male	Non-small cell lung cancer	2020	Standard
Q13	Patient	Hull	Male	Lung cancer	2022	Standard
Q14	Patient	London	Male	Non-small cell lung cancer	2022	Non-typical

### Data analysis

Initial coding was conducted inductively, to allow themes to emerge directly from participants' accounts (26). This approach ensured that the analysis remained grounded in the data and was not constrained by predetermined categories. Following this, the Consolidated Framework for Implementation Research (CFIR) was applied deductively as an organising framework to structure and interpret the emergent themes (27). Using CFIR in this way enabled the alignment of inductive insights with established implementation constructs, facilitating systematic identification of barriers and facilitators to AI adoption while preserving the richness of participants' perspectives.

The analysis was conducted by three researchers (MC, HS and CS), who are social scientists, have no clinical background or lived experience and were not involved in the main trial. This approach allowed the interpretation of the data to be free of personal or professional influences. The results were then discussed with all authors before they were finalised.

### Ethics

Ethics approval was obtained from the Bayes Business School Ethics Review Committee at City St George's University of London (Ethics ETH2223-0030). All participants were given an information sheet and gave written consent prior to participating to the study.

## Results

**Figure 1:**
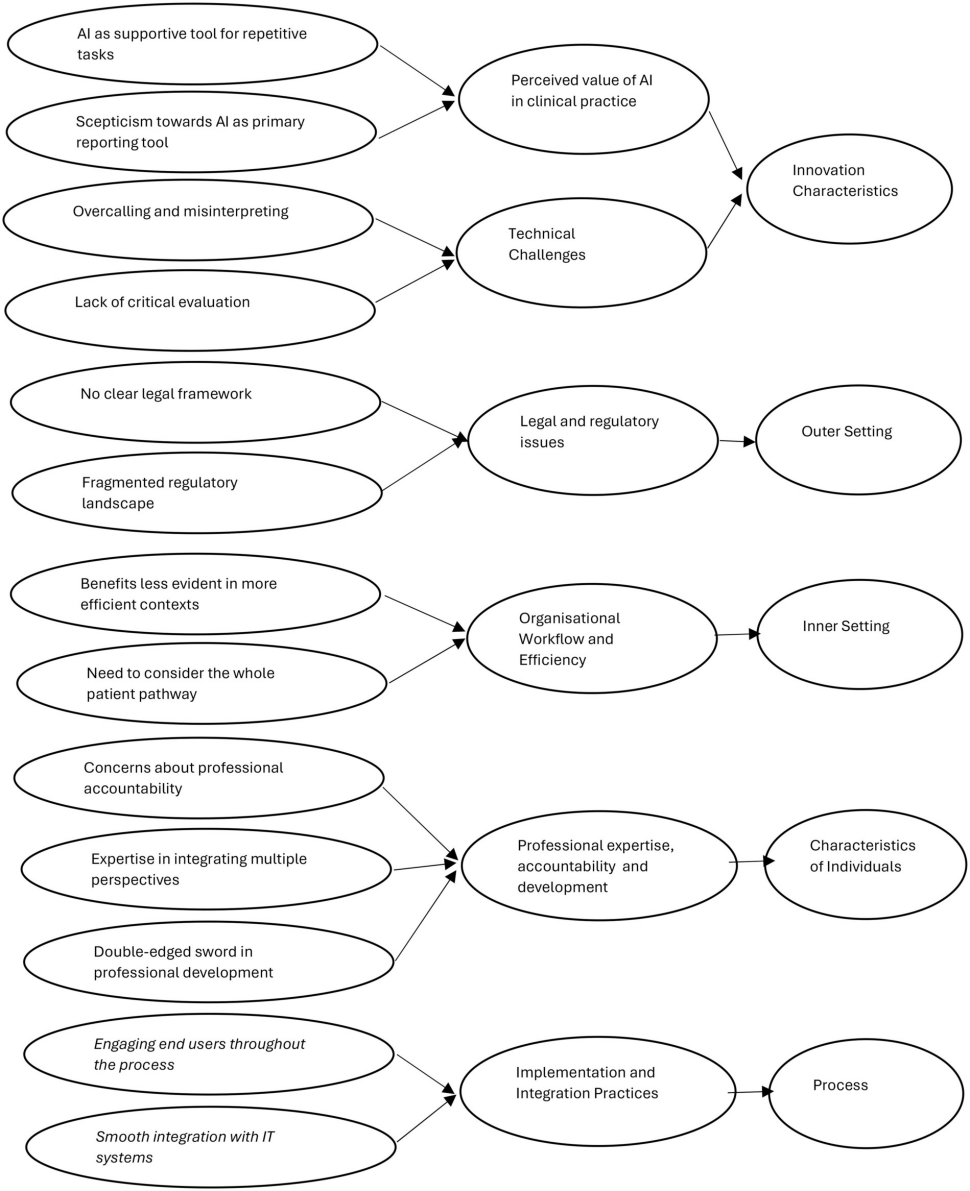
Themes and subthemes within the CFIR framework.

Initial themes and subthemes that emerged from our analysis are presented here under the five domains of the CFIR framework: interventions characteristics, outer setting, inner setting, characteristics of individuals and process. The themes are summarised in Figure 1.

### Domain 1: intervention characteristics

Within the first domain of CFIR, that concerns intervention characteristics, two main themes were identified: the perceived value of AI in clinical practice and its technical challenges. Overall, healthcare professionals and PPIE participants saw the value of the AI tool in supporting professionals in completing repetitive tasks but were very sceptical about the use of AI as a primary reporting tool. This was mostly due to the technical challenges that the technology still faces, particularly the issue of overcalling and the lack of a critical approach to evaluating images. Below we expand on these themes and subthemes in more detail.

#### Theme: Perceived value of AI in clinical practice

##### Subtheme: AI as a supportive tool for repetitive tasks

AI was seen as a supportive tool, providing an additional layer of support to radiographers and radiologists for triaging and guidance. In this role, the technology was perceived as offering reassurance and validation by cross-checking findings, highlighting potential oversights, and reducing the likelihood of human error. Clinicians emphasised that such support served as a safety net. This positioned AI as a secondary, complementary resource that could enhance accuracy in the diagnostic process.

“I think that's what you foresee your AI doing … certainly for the immediate future is being a second read of everything, because it is that potential to pick up something that is there that you would walk past because you get a phone call midway through reading a case or you're distracted, you're thinking about lunch, or whatever, that it's there and it flags it up”. (P23 Consultant Radiologist, Site 5)

Radiographers and radiologists saw the value of the AI tool in supporting them with tasks that were repetitive and more mechanically done. These tasks, though integral to clinical practice, are often viewed as detracting from the more specialised and interpretative aspects of their roles. By assigning such mechanical duties to AI, professionals could devote greater attention to complex image interpretation, clinical judgment, and patient interaction.

“…. computers are good at doing repetitive jobs without getting involved, without getting tired. So, I might get a phone call from my wife and then you can imagine the report going downhill”. (P12, Consultant radiologist, Site 3)

##### Subtheme: Scepticism towards AI as a primary reporting tool

Both healthcare professionals and PPIE members perceived AI more as a second pair of eyes that supports the work of professionals, rather than an autonomous decision-making tool. Healthcare professionals raised concerns that the technology was not yet advanced enough to be trusted as a sole reader, while patients and carers in particular expressed a clear desire for human interaction, especially when news could be distressing.

“I think it's preferable to have a human being break the news to you as against a machine or even a letter. I got the letter that said “blah de blah, your hospital will be in touch”. Obviously, my first thought was it's lung cancer. They've scanned the lungs, that's what it must be, but yes, I'd prefer it to come from a human being”. (Q13, patient, male)

#### Theme: Technical challenges

##### Subtheme: Overcalling and misinterpreting

A potential drawback of integrating AI into clinical decision-making according to healthcare professionals is the likelihood of the tool to overcall abnormalities, leading to more false positives and ultimately increasing the frequency of CT scan referrals. While such caution may reduce the risk of missed diagnoses, it may also contribute to longer diagnostic pathways and higher healthcare costs. Thus, careful calibration of AI recommendations and the development of guidelines for their clinical interpretation are essential to balance patient safety with resource efficiency.

“I suppose the disadvantages of that is that you might see a spike in number of CT referrals because we're erring on the side of caution”. (P15, Consultant Radiographer, Site 3)

##### Subtheme: Lack of critical evaluation

Another limitation of the AI tool according to radiologists and radiographers is that, as it currently stands, the tool is unable to critically evaluate the image and go beyond the actual image, therefore limiting their analysis and narrowing their diagnostic role. Healthcare professionals proceed with the diagnosis and reporting based on not only the x-ray but also other clinical characteristics of the patients and previous x-rays which help them interpret the findings of the image.

“It's not just the x-ray in isolation. It's also looking at the clinical information, the ethnicity of the patient, the age of the patient, and then forming a whole picture about what I might be seeing, whereas your tool is just saying there's a blob there. […] it's context and having a little bit of knowledge about the whole picture, rather than just looking at a 2D image”. (P22, Consultant Radiologist, Site 4)

### Domain 2: outer setting

When considering the outer setting, CFIR's second domain, the main theme developed was around the lack of clear legal and regulatory frameworks.

#### Theme: Legal and regulatory issues

##### Subtheme: No clear legal framework

Concerns were raised regarding the absence of a clear legal framework that accounts for the integration of AI tools in medical decision-making processes. While current regulatory frameworks in the UK do not permit the use of AI as a sole reader in clinical settings, participants felt that the legal system has yet to adequately address questions of liability, and oversight. This gap generates uncertainty for healthcare professionals, patients and their carers. For instance, if an AI system contributes to a misdiagnosis or adverse outcome, it remains unclear whether liability should fall on the physician who relied on the tool or the institution that adopted it. This uncertainty is likely to impact on healthcare professionals' behaviour as stated by the consultant radiologist below:

“… this is the emerging legal thing, is where does the liability sit? Because the AI companies can't take the liability for being wrong. You have to still have oversight of it”. (P26, Consultant Radiologist, Site 6)

##### Subtheme: Fragmented regulatory landscape

Participants often discussed the regulatory burden and the lack of coordinated guidance within the digital health ecosystem. Multiple organisations are involved, including the Medicines and Healthcare products Regulatory Agency (MHRA), the primary regulator for digital devices responsible for ensuring safety and effectiveness; The National Institute for Health and Care Excellence (NICE)**,** which ensures interventions are cost-effective; and NHS England, which oversees the digital transformation of the NHS. Participants noted that each body provides its own guidance, some of which may be outdated, creating confusion and challenges for stakeholders navigating regulatory requirements.

“Yes, the regulations are a nightmare. The MHRA are looking after it because it's a digital device. Then, everyone has an opinion on, the RCR have got some stuff. The BIR have got some stuff. NHS England has got some stuff. NHSX has got something else. Then, some stuff that was written three years ago is no longer relevant”. (P23, Consultant Radiologist, Site 5)

At the same time, the lack of regulation and reimbursing mechanisms was raised by the participants. As one radiologist put it:

“It's like NICE—you need NICE for AI—bang for your buck”. (P23, Consultant Radiologist, Site 5)

### Domain 3: inner setting

CFIR's inner setting captures the organisational context that shapes the implementation of AI in healthcare and can determine how well AI aligns with local priorities and professional practices. In our study, participants talked about organisational workflow and efficiencies, highlighting how benefits are less evident in more efficient contexts and that AI needs to consider the wider patient pathway in which it is implemented.

#### Theme: Organisational workflow and efficiency

##### Subtheme: Benefits less evident in more efficient contexts

Organisational context played a significant role in how healthcare professionals understand the efficiencies the AI tool would bring. Site 2 is a radiology department within an urban teaching hospital, where patients referred for radiographic imaging by their general practitioner (GP) are able to attend on a walk-in basis without the need for a scheduled appointment. The department employs 13 reporting radiographers, five of whom are specifically responsible for interpreting GP-referred radiographs. Owing to the relatively large workforce, the department has been able to maintain efficient service delivery, including the provision of same day CT scans for patients with abnormal radiographic findings, even prior to the implementation of the AI tool. Staff acknowledged, however, that the AI tool may demonstrate greater utility in departments where workforce limitations present a barrier to timely service provision. As an example, Site 2 already offers same day CT scans, suggesting the new tool will have no significant impact on reducing the time to diagnosis.

##### Subtheme: Need to consider the whole patient pathway

Both PPIE participants and healthcare professionals suggested that the implementation of AI cannot be seen in isolation from the whole patient pathway. When patients and carers talked about their personal journey to lung cancer diagnosis, it became apparent that delays in the process were often seen earlier and unless wider structural issues were considered, there was little benefit from AI. As mentioned by patient Q4 below, most patients reported delays in receiving a referral from the GP, who would disregard initial symptoms.

“… you still need to get the GP to refer you, and what we heard today is, sometimes the GPs don't refer you when they should have done. They can actually think you don't have cancer and you don't need an x-ray. I think, was it […] said, it was stress, but it's not stress, it's cancer, but you'll never get the benefit of the AI unless the doctor actually sends you for that x-ray”. (Q4, patient, male)

Equally, healthcare professionals highlighted that little benefit would be seen from faster reporting of x-rays if there were no available CT scans in their organisation. A radiologist from Site 5 talked about the lack of capacity to do same-day CT scans and the domino effect this might have further down the line:

“It's—you squeeze a balloon. It's fine—we will do them in the end. We are doing them now—we're just doing them two or three days down the line. It's if we're doing those now, what are we not doing? You're causing delays in something else”. (P22, Consultant Radiologist, Site 5)

### Domain 4: characteristics of individuals

CFIR's fourth domain considers the knowledge, beliefs, and personal attributes of those involved in implementation.

#### Theme: Professional expertise, accountability and development

##### Subtheme: Expertise in integrating multiple perspectives

Professionals look “at the patient holistically in a way that the AI can't”. By viewing AI as auxiliary to their work, they feel it does not directly replicate the work or professional expertise of those involved and therefore does not pose a threat to their established roles or competencies.

Similarly, patients agreed that the AI can work in the background but still there is an important element of trust in the doctor-patient relationship, that goes beyond the simple reporting of an image.

“We still trust our doctors, by and large. I'm constantly being asked at my consultations whether I mind the—well, I'm just thinking of all the consultations and investigations I've had in the last two or three months. On every one, there's been someone learning, someone watching, someone sitting in. I'm always asked, or I'm told that so-and-so is learning today: “Do you have…?” Some people would call me naive, but I always accept that people need to learn and I trust that they've gone through the correct procedures”. (Q12, Patient, Male)

##### Subtheme: Concerns about professional accountability

During the training that all participants undertook before the AI tool was implemented, it was highlighted that the intended use of the AI was as a clinical decision support tool, and that they retained responsibility for the accuracy and completeness of the report. Still, accountability appears to be a primary factor influencing radiographers' tendency to adopt a cautious approach by aligning with the AI tool's recommendations when abnormalities are flagged, even in cases where they suspect a false positive. Given that radiographers remain ultimately responsible for the clinical decisions they make, they are inclined to prioritise risk mitigation over independent judgement in such scenarios.

These concerns were also raised by PPIE participants, who see AI as a “black box”, unable to even distinguish when an error has occurred:

“So when it makes a mistake, how will you know it's made a mistake?” (Q4, Patient, Male)

##### Subtheme: Double-edged sword in professional development

Conflicting perspectives emerged regarding the extent to which the tool might facilitate or impede the professional development of healthcare practitioners, particularly junior colleagues. On one hand, participants recognised the potential value of AI as a supportive aid during the learning process. From this perspective, the tool was seen as a useful adjunct to training, offering reassurance to less experienced staff as they built their knowledge and skills. Conversely, concerns were also raised that reliance on AI could undermine the development of independent clinical judgement, leading junior practitioners to defer to algorithmic outputs rather than cultivating confidence in their own assessments. Such dependence, it was argued, might ultimately diminish critical thinking skills and reduce trust in professional expertise. These divergent views were expressed consistently across both radiographers and radiologists, highlighting a wider uncertainty about the long-term implications of AI integration for workforce development and professional identity.

“If you're a junior registrar and the AI tells you something is wrong, you might—which is one of the funny things about it, about training, is how these junior radiologists are now going to be trained with them being the expert. But then if you want a radiologist that's only been trained their entire career and has AI in the background telling them what's what, then how comfortable they are overriding the AI?” (P23, Consultant Radiologist, Site 5)

### Domain 5: process

#### Theme: Implementation and integration practices

##### Subtheme: Engaging end users throughout the process

Company staff talked about the importance of engaging with end users, i.e., healthcare professionals and IT staff from the hospitals, throughout the process. From finalising the protocol prior to the implementation of the tool, to training radiologists and radiographers and working closely with them to receive feedback, it was clear this engagement was key to ensure a smooth transition to the new system.

“So the first couple of weeks of the implementation of the deployment are very critical for us, where the client partner would work very closely with the user to make sure that they are able to integrate or embed the use of the software within their daily practice”. (I6, Impact measurement)

“Once the deployment is done, we need the radiologist to—we need to train the radiologists and the consultant radiographers to actually tell them how to use the system effectively, and whatever—if they face any problems on a day-to-day basis, we should be there to help them”. (I2, Client Success)

##### Subtheme: Smooth integration with IT systems

Despite AI being presented as a novel technology, the integration of the tool did not pose significant challenges to IT teams that were involved in its implementation:

“Yes, I mean it seemed on the whole fairly smooth really. I wouldn't say it was more problematic than a lot of IT projects”. (P10, Senior Clinical Systems Designer, Site 2)

The main reason for that was that the tool was not trained using patient data from the sites where it was being implemented. It was used to identify abnormalities and the system reported on whether the tool identified one or not:

“Is there an abnormality or not? If the answer will be a one for yes and a zero for no. What we did was file that to an order question that's attached to that particular chest x-ray that just shows a yes or a no, and then all we do is build a column that corresponds to that question. That's literally all it is. There's nothing specific … It's not any different to any other workflow that we use that looks at a specific order question”. (P10, Senior Clinical Systems Designer, Site 2)

## Discussion

In addressing the translation of AI from development to its implementation in practice, our study focuses not only on the technology itself, but also on the inner and outside context in which these technologies operate, the individuals involved as well as the implementation processes.

Overall, both professional and PPIE groups held a positive view of AI but highlighted a number of issues related to its implementation. PPIE participants perceived AI as a means of speeding the time to diagnosis and potentially making the diagnosis more accurate. Yet, they mostly saw AI as a second pair of eyes that supported the work of professionals, rather than an autonomous decision-making tool and they expressed a clear desire for human interaction, especially when potentially distressing news has to be broken. We also found that professionals' responses to the implementation and use of the tool were highly influenced by the way in which its functionality was integrated into existing workflow as well as its perceived value. The tool's role as a new triaging tool, rather than as a second reader, meant that it was not directly emulating the work and expertise of the professionals involved and hence did not impose a threat to their existing roles and expertise. At the same time, the workflow contexts of the different sites also created variations in staff responses; professionals at sites where workload pressures were relatively light perceived the triaging functionality of the AI tool as providing relatively little added value to their work. Finally, lack of clear governance in implementing AI tools in the NHS was one of the main barriers innovators faced when introducing their tool in practice. Company staff also highlighted the importance of engaging with end users throughout the process as key in the successful deployment of the tool.

Previous literature has shown there are mixed experiences among healthcare professionals, patients and the public regarding AI implementation, emphasising the need for further research to address these perspectives (10, 28). Although AI is being introduced into many aspects of our lives, the debate on what professionals and the public think of it is particularly intense in the healthcare arena. This is not only because professional groups exert such control over healthcare services, but also because the exercise of diagnostic decision-making and judgement in this arena brings with it much higher stakes for all concerned (29). Decisions are being made about patients, not clients, and individual professionals are personally accountable for those decisions (30).

Our findings show that healthcare professionals maintain their expertise and view AI tools as complementary to their role. As Davenport and Kalakota (31) note, “radiologists do more than read and interpret images”. They also, inter alia, relate findings from images to other medical records and test results and collaborate with professional colleagues and consult with them on diagnosis and treatment. Moreover, the technical challenges faced by AI tools probably contribute to the idea that healthcare professionals know better, not least because AI lacks prior knowledge. Katal et al. (32) confirm that AI's failure to consider clinical data and prior imaging can lead to errors in the interpretation and reporting of images. Our study confirms this is what professionals believe.

Our study also aligns with previous studies on patients' views (13), confirming that patients and their carers are positive about the use of AI in diagnostic pathways, as long as they act as a supporting tool rather than a first reader. As a study by the Health Foundation showed “the public strongly value keeping a human in the loop for many uses of AI in health care” (28). Human oversight was shown as key in a report by the Royal College of Radiologists, that also showed how public acceptance of AI in radiology is strongly influenced by familiarity with AI (33). Our study did not confirm these findings, but a discrete choice experiment that involved two co-authors showed that patients and carers who have had a lung cancer diagnosis showed a stronger preference than members of the public for results processed through a combination of human and AI input rather than by humans alone (34).

Our study is not without limitations. It is based on interviews with clinicians to explore their experiences, rather than ethnographic observations while they are performing their tasks. We also wanted to conduct interviews with two sites that were initially meant to be part of the deployment but decided not to participate to understand why this was the case. But this was not possible as ethics approval was obtained to visit only those sites that deployed the AI tool.

### Policy implications

The adoption of AI innovation in healthcare is complex and the case we analysed in this study is not an exception. NICE, in its Early Value Assessment of AI-derived software for chest x-rays for suspected lung cancer in primary care referrals, concludes that the current evidence is insufficient to justify its routine use in the NHS and recommends that “access to the technology should be through company, research or non-core NHS” (16). NICE call for more evidence not only on the accuracy of such tools and their impact on costs and resource use, but also on “patient perceptions of using AI-derived software”. Our study sheds more light in this respect.

Our findings have direct policy and practice implications beyond the English NHS. AI innovations are not off the shelf products. Policy makers, industry and healthcare practitioners will need to address the implementation challenges of introducing AI in practice if they are to facilitate consistent adoption at scale.

## Data Availability

The datasets presented in this article are not readily available because they consist of qualitative interview transcripts containing sensitive and potentially identifiable information. In order to protect participant confidentiality and privacy, the data cannot be made publicly available. Requests for further information should be directed to the corresponding author.

## References

[1] Department for Science, Innovation and Technology. AI Opportunities Action Plan. London: Crown (2025). Available online at: https://www.gov.uk/government/publications/ai-opportunities-action-plan/ai-opportunities-action-plan

[2] NHS England Transformation Directorate. Artificial Intelligence (AI) Funding Streams (2025). Available online at: https://transform.england.nhs.uk/ai-lab/explore-all-resources/understand-ai/artificial-intelligence-ai-funding-streams/ (Accessed September 10, 2025).

[3] Alowais SA Alghamdi SS Alsuhebany N Alqahtani T Alshaya AI Almohareb SN Revolutionizing healthcare: the role of artificial intelligence in clinical practice. BMC Med Educ. (2023) 23(1):689. 10.1186/s12909-023-04698-z 37740191 PMC10517477

[4] Kuo RYL Harrison C Curran T-A Jones B Freethy A Cussons D Artificial intelligence in fracture detection: a systematic review and meta-analysis. Radiology. (2022) 304(1):50–62. 10.1148/radiol.211785 35348381 PMC9270679

[5] Pinto-Coelho L. How artificial intelligence is shaping medical imaging technology: a survey of innovations and applications. Bioengineering. (2023) 10(12):1435. 10.3390/bioengineering10121435 38136026 PMC10740686

[6] Crosby D Bhatia S Brindle KM Coussens LM Dive C Emberton M Early detection of cancer. Science. (2022) 375(6586):eaay9040. 10.1126/science.aay9040 35298272

[7] UK Parliament Communications and Digital Committee. UK Risks Becoming an ‘Incubator Economy’ If We Don’t Take Action to Support Our Tech Companies to Scale up (2025) Available online at: https://committees.parliament.uk/committee/170/communications-and-digital-committee/news/205059/uk-risks-becoming-an-incubator-economy-if-we-dont-take-action-to-support-our-tech-companies-to-scale-up/ (Accessed September 10, 2025).

[8] Car J Sheikh A Wicks P Williams MS. Beyond the hype of big data and artificial intelligence: building foundations for knowledge and wisdom. BMC Med. (2019) 17(1):143. 10.1186/s12916-019-1382-x 31311603 PMC6636050

[9] Kelly CJ Karthikesalingam A Suleyman M Corrado G Dominic K. Key challenges for delivering clinical impact with artificial intelligence. BMC Med. (2019) 17(1):195. 10.1186/s12916-019-1426-2 31665002 PMC6821018

[10] Lawrence R Dodsworth E Massou E Sherlaw-Johnson C Ramsay AIG Walton H Artificial intelligence for diagnostics in radiology practice: a rapid systematic scoping review. eClinicalMedicine. (2025) 83:103228. 10.1016/j.eclinm.2025.103228 40474995 PMC12140059

[11] Emanuel EJ Wachter RM. Artificial intelligence in health care: will the value match the hype? JAMA. (2019) 321(23):2281–82. 10.1001/jama.2019.4914 31107500

[12] Chen Y Stavropoulou C Narasinkan R Baker A Scarbrough H. Professionals’ responses to the Introduction of AI innovations in radiology and their implications for future adoption: a qualitative study. BMC Health Serv Res. (2021) 21(1):813. 10.1186/s12913-021-06861-y 34389014 PMC8364018

[13] Gundlack J Thiel C Negash S Buch C Apfelbacher T Denny K Patients’ perceptions of artificial intelligence acceptance, challenges, and use in medical care: qualitative study. J Med Internet Res. (2025) 27(1):e70487. 10.2196/70487 40373300 PMC12123243

[14] Ramsay AIG Crellin N Lawrence R Walton H Bagri S Dodsworth E Procurement and early deployment of artificial intelligence tools for chest diagnostics in NHS services in England: a rapid, mixed method evaluation. eClinicalMedicine. (2025) 89:103481. 10.1016/j.eclinm.2025.103481 41357329 PMC12675025

[15] Farič N Hinder S Williams R Ramaesh R Bernabeu MO van Beek E Early experiences of integrating an artificial intelligence-based diagnostic decision support system into radiology settings: a qualitative study. J Am Med Inform Assoc. (2023) 31(1):24–34. 10.1093/jamia/ocad191 37748456 PMC10746311

[16] NICE. Artificial Intelligence-Derived Software to Analyse Chest x-Rays for Suspected Lung Cancer in Primary Care Referrals: Early Value Assessment. London: NICE (2025).

[17] NICE. Overview | Artificial Intelligence (AI) Technologies to Help Detect Fractures on x-Rays in Urgent Care: Early Value Assessment. London: NICE (2025). Available online at: https://www.nice.org.uk/guidance/hte20

[18] NICE. 1 Recommendations | Artificial Intelligence (AI)-Derived Software to Help Clinical Decision Making in Stroke. London: NICE (2024). Available online at: https://www.nice.org.uk/guidance/dg57/chapter/1-Recommendations

[19] Baldwin D. (2023). Does Triage of Chest x-Rays with Artificial Intelligence Shorten the Time to Lung Cancer Diagnosis: A Randomised Controlled Trial. ISRCTN Registry. 10.1186/ISRCTN78987039

[20] Kumar A Patel P Robert D Kumar S Khetani A Reddy B Accuracy of an artificial intelligence-enabled diagnostic assistance device in recognizing normal chest radiographs: a service evaluation. BJR Open. (2023) 6(1):tzae029. 10.1093/bjro/tzae029 PMC11441651 39350939

[21] Robert D Sathyamurthy S Singh AK Matta SA Tadepalli M Tanamala S Effect of artificial intelligence as a second reader on the lung nodule detection and localization accuracy of radiologists and non-radiology physicians in chest radiographs: a multicenter reader study. Acad Radiol. (2025) 32(3):1706–17. 10.1016/j.acra.2024.11.003 39592384

[22] Khan FA Majidulla A Tavaziva G Nazish A Abidi SK Benedetti A Chest x-ray analysis with deep learning-based software as a triage test for pulmonary Tuberculosis: a prospective study of diagnostic accuracy for culture-confirmed disease. Lancet Digit Health. (2020) 2(11):e573–81. 10.1016/S2589-7500(20)30221-1 33328086

[23] NHS England. Running focus groups for patient and public engagement (2016). Available online at: https://www.england.nhs.uk/wp-content/uploads/2016/07/bitesize-guide-focus-groups.pdf (Accessed September 10, 2025).

[24] Lammons W Silkens M Hunter J Shah S Stavropoulou C. Centering public perceptions on translating AI into clinical practice: patient and public involvement and engagement consultation focus group study. J Med Internet Res. (2023) 25:e49303. 10.2196/49303 37751234 PMC10565616

[25] NIHR. Guidance for Applicants on Working with People and Communities (n.d.). Available online at: https://www.nihr.ac.uk/research-funding/application-support/working-with-people-and-communities (Accessed September 10, 2025).

[26] Thomas DR. A general inductive approach for analyzing qualitative evaluation data. Am J Eval. (2006) 27(2):237–46. 10.1177/1098214005283748

[27] Damschroder LJ Reardon CM Opra Widerquist MA Lowery J. The updated consolidated framework for implementation research based on user feedback. Implement Sci. (2022) 17(1):75. 10.1186/s13012-022-01245-0 36309746 PMC9617234

[28] The Health Foundation. AI in Health Care: What Do the Public and NHS Staff Think? London: The Health Foundation (2024). Available online at: https://www.health.org.uk/reports-and-analysis/analysis/ai-in-health-care-what-do-the-public-and-nhs-staff-think

[29] Challen R Denny J Pitt M Gompels L Edwards T Tsaneva-Atanasova K. Artificial intelligence, bias and clinical safety. BMJ Qual Saf. (2019) 28(3):231–37. 10.1136/bmjqs-2018-008370 30636200 PMC6560460

[30] He J Baxter SL Xu J Xu J Zhou X Zhang K. The practical implementation of artificial intelligence technologies in medicine. Nat Med. (2019) 25(1):30–6. 10.1038/s41591-018-0307-0 30617336 PMC6995276

[31] Davenport T Kalakota R. The potential for artificial intelligence in healthcare. Future Healthc J. (2019) 6(2):94–8. 10.7861/futurehosp.6-2-94 31363513 PMC6616181

[32] Katal S York B Gholamrezanezhad A. AI in radiology: from promise to practice−A guide to effective integration. Eur J Radiol. (2024) 181(December):111798. 10.1016/j.ejrad.2024.111798 39471551

[33] The Royal College of Radiologists. The Future of AI in Healthcare: Public Perceptions of AI in Radiology (2025). Available online at: https://www.rcr.ac.uk/media/poelyzlz/rcr-reports-the-future-of-ai-in-healthcare-public-perceptions-of-ai-in-radiology.pdf (Accessed September 10, 2025).

[34] Banerjee A Rawlinson J Woznitza N David BR. Results from a Choice-Based Conjoint Experimental Study on Patients’ Preferences for Chest x-Ray Results Processing and Communications When Referred by Their GP/ Primary Care Doctor in NHS, England. SSRN Scholarly Paper 5533060. Social Science Research Network (2024). 10.2139/ssrn.5533060

