# Supplementary material for: Implementing AI innovation in radiology departments in the English NHS: a qualitative study on the experiences of professionals, patient groups and innovators
Source: Front Digit Health. 2026 Mar 17;8:1736911. doi: 10.3389/fdgth.2026.1736911 (PMC13036200; doi:10.3389/fdgth.2026.1736911)
Supplement: Supplementary file 1 [file Datasheet1.docx]

**SUPPLEMENTARY MATERIAL**

**Interview schedule**

***Pre-implementation phase***

*Developer staff*

1. How have you been involved in the development of the AI tool in terms of expertise, management etc.?
2. What do you see as the key innovative features of the tool and how were they developed?
3. What are the major risks and benefits attached to its use in practice?
4. What knowledge do you have of the healthcare environment in the UK?
5. What challenges do you think this may create for the implementation of the tool?
6. How do you envisage the tool being deployed in practice, and what are the implications for hospital staff and patients respectively?

*Hospital staff*

1. Could you say a little bit about yourself in terms your job, organisational role, professional background and experience?
2. Please talk me through your role in the current pathway for lung cancer diagnosis. How and when do you interact with other staff and with patients, and what information is exchanged at these points.
3. To what extent is your hospital already implementing the National Optimal Lung Cancer Pathway (NOLCP)? If not, what do you see as the constraints on doing so?
4. Regarding the AI tool, what contact or communications have you had with the development team, and do you have any previous experience of the use of other AI-based tools in your work?
5. How do you acquire knowledge on new AI-based tools in your field generally, and the tool specifically? Do you have sufficient knowledge and awareness to be ready for its introduction?

***Implementation phase***

*Developer staff*

1. Tell me about your role in implementing the AI tool in this hospital.
2. What stage of implementation has been achieved so far?
3. What changes have been made within the hospital to enable the effective deployment of the tool?
4. What are the implications for the pathways and workflows around lung cancer diagnosis?
5. How have these changes affected the experience of staff and patients?
6. What were the major challenges in implementing the tool and how were they overcome?
7. What do you see as the key learning points from the implementation so far?

*Hospital staff*

1. From your experience, how does the AI tool change the patient pathway in practice?
2. What do you see as the major impacts of the use of the AI tool on the way in which your work is carried out, including your collaboration with colleagues? What do you see as the benefits and concerns related to this impact, e.g. accuracy of diagnosis, speed, greater reliability, etc. ?
3. Has the use of this tool affected the way you interact with patients?
4. Were there any barriers to your ability to use this tool effectively (e.g. in terms of workflow)?
5. How has the use of this tool affected your own role and skill-set?
6. How confident are you in the results provided by AI tool?

**FOCUS GROUP SCHEDULE FOR PPIE**

**Welcome and introductions (CS)**

CS to welcome everyone and give a brief overview of what the project is about and what is expected from the participants.

CS to stress that participants should only share as much or as little as they want of their personal experiences and stories. We recognise that these discussions may trigger difficult emotions and reassure everyone they can stop if they don’t feel like. We can always signpost them to relevant groups if they need support. We hope that by discussing personal stories with others this can help other two.

CS to ask everyone if recording is fine with them – reassuring anonymity.

Keep introductions short (name, carer or patient, how long since experience of x-ray). Remind them they will have an opportunity to talk about their experience when we do the break-out rooms.

BREAK OUT ROOMS

**Overall views on AI:**

- What are your thoughts on the use of AI in healthcare?
- What are your thoughts on the use of AI as a diagnostic tool, such as the use of AI in detecting abnormalities in chest x-rays?

**Personal experiences of the pathways**

- When you think of your experience back when you, or the person you cared for, had to do an x-ray for suspected lung cancer, what were the things that mattered to you the most?
- What did you expect? How much information did you have prior to the x-ray?
- Who broke the news to you (whether good or bad)
- How long did you have to wait for the results? What happened next?

**Specific issues related to the project**

- How do you feel about AI being used in radiology?
- How much do you think people should know about the involvement of AI?
- The tool aims to reduce the time of reporting x-rays and potentially even offer same-day CT-scan. What are your thoughts about that?
- Any other thoughts?

MAIN ROOM

CS to explain next steps.

CS to ask participants whether they would like to be kept informed about the findings and whether they would be happy to participate in dissemination activities

THANK everyone!
